# Review of the Relationships Between Human Gut Microbiome, Diet, and Obesity

**DOI:** 10.3390/nu16233996

**Published:** 2024-11-22

**Authors:** Ondřej Patloka, Tomáš Komprda, Gabriela Franke

**Affiliations:** Department of Food Technology, Mendel University in Brno, 61300 Brno, Czech Republic; xpatlok3@mendelu.cz (O.P.); gabriela.franke@mendelu.cz (G.F.)

**Keywords:** secondary bile acids, short-chain fatty acids, butyrate, dietary fiber, resistant starch, intestinal barrier function, immune homeostasis

## Abstract

Obesity is a complex disease that increases the risk of other pathologies. Its prevention and long-term weight loss maintenance are problematic. Gut microbiome is considered a potential obesity modulator. The objective of the present study was to summarize recent findings regarding the relationships between obesity, gut microbiota, and diet (vegetable/animal proteins, high-fat diets, restriction of carbohydrates), with an emphasis on dietary fiber and resistant starch. The composition of the human gut microbiome and the methods of its quantification are described. Products of the gut microbiome metabolism, such as short-chain fatty acids and secondary bile acids, and their effects on the gut microbiota, intestinal barrier function and immune homeostasis are discussed in the context of obesity. The importance of dietary fiber and resistant starch is emphasized as far as effects of the host diet on the composition and function of the gut microbiome are concerned. The complex relationships between human gut microbiome and obesity are finally summarized.

## 1. Introduction

The most general definition of obesity is a BMI (Body Mass Index; weight in kg divided by the square of height in m) of over 30 kg/m^2^ [1]. Obesity is a consequence of the effects of multiple factors including genetics, excessive energy intake (high-fat diets, high carbohydrate diets, excessive alcohol intake), sedentary lifestyle, socioeconomic status, poor sleep quality, parental weight, or environment [2]. It is a complex disease that increases the risk of many other pathologies such as ischemic heart disease, stroke, type 2 diabetes (T2D), hypertension, hyperlipidemia, as well as several types of cancer [3]. According to Strohacker et al. [4], there is no effective, well-defined, evidence-based intervention for preventing obesity. The authors [4] mention long-term negative health consequences of weight cycling, compare the risks of weight change pattern with obesity maintenance, and discuss physiological alterations during weight loss that promote weight regain and the effect weight regain has on the rate and type of accumulation of adipose tissue. Attempts to maintain weight loss long-term, including lifestyle interventions aiming to decrease energy intake and increase physical activity, are mostly unsuccessful [5]. Nevertheless, due to its epidemic proportions (the current worldwide prevalence in women and men is nearly 15% and nearly 11%, respectively), the treatment of obesity is of an utmost importance [6].

Another potential obesity modulator being considered is gut microbiome [6,7,8,9]. In this subject, review articles are available regarding general aspects of the gut microbiota [10], its principal metabolites, such as short-chain fatty acids (SCFAs; [11,12,13]) and bile acids (BAs; [14,15,16]), and dietary components with the potential to modulate the gut microbiome, such as dietary fiber (DF; [17,18] or resistant starch (RS; [19]). SCFAs are organic acids, predominantly produced within the anaerobic fermentation of indigestible food components by the gut microbiota [20], able to modulate production of the host appetite-regulating hormones and energy intake [21]. The present study summarizes the principal findings described in more detail in the above-mentioned articles, with the objective of indicating possible ways to prevent obesity via DF/RS intake and modulation of the gut microbiome composition, including its metabolites SCFAs and BAs.

## 2. Human Gut Microbiome Composition

Based on the analysis of the human gut microbiome, the most frequently represented phyla here are Bacteroidetes (synonym Bacteroidota) and Firmicutes (synonym Bacillota), which comprise 90% of all human gut phyla; the remaining 10% represent Actinobacteria (synonym Actinomycetota), Proteobacteria, Fusobacteria, and Verrucomicrobia [10].

Firmicutes are constituted mostly by Gram-positive genera with *Clostridium* being the most abundant genera (95%), followed by *Lactobacillus*, *Bacillus*, *Enterococcus*, and *Ruminococcus* [22]. Additionally, Bacteroidetes are Gram-negative bacteria with the most abundant genera being *Bacteroides* and *Prevotella* followed by *Parabacteroides* and *Alistipes* [23]. As far as Actinobacteria are concerned, the main representative on the genus level is *Bifidobacterium* [24].

The following factors can influence an individual’s gut microbial composition during the duration of life: being preterm or full-term born; vaginal or caesarean delivery; nursing by human milk or a substitute [25,26,27]; BMI; exercise; diet [22,25,28,29]; and past illnesses including the use of drugs and antibiotics [30]. Following the metagenomic data of Rothschild et al. [31], it has been shown that diet and environment affect the composition of the human gut microbiome in healthy individuals more than host genetics.

Moreover, the question of the stability of the human gut microbiome in adult individuals is still not fully resolved. Some studies identified three distinct clusters of balanced microbial states called enterotypes, dominated by Bacteroides (enterotype I), Prevotella (enterotype II), and Ruminococcus (enterotype III), respectively. The bacterial communities of the different enterotypes can possibly generate energy by either fermentation of carbohydrates (enterotype I) or from mucin glycoproteins (enterotypes I and III) and can also differ regarding level of vitamin production [24]. On the other hand, the data of Jeffery et al. [32] or Knights et al. [33] suggest the presence of continuous gradients of variation in bacterial communities rather than discrete enterotypes.

More than 90% of gut bacteria cannot be detected by conventional culture methods [34]. However, the relative abundance of bacterial groups does not always correspond to the absolute abundance of bacterial taxa observed. 16S rRNA gene sequencing [35] and whole genome sequencing [36,37,38,39] are currently two main approaches used in the species and functional analysis of gut microbiome.

## 3. Products of the Gut Microbiome Metabolism

According to current knowledge, it is estimated that the intestinal microbiota includes up to 100 trillion microbial cells and contains more than 22 million microbial genes encoding many enzymes with diverse metabolic activity [40]. Intestinal microbiota can produce a large number of bacterial metabolites with a wide spectrum of biological activities for the host organism. These metabolites can be generally divided into three types: (1) metabolites produced by bacterial degradation of nutrients and other food components from a diet, such as SCFAs; (2) metabolites created by the host organism and modified by microbiota, such as secondary BAs; (3) metabolites produced de novo by the microbiota, such as lipopolysaccharides (LPSs; [41]). The present article focuses especially on SCFAs and BAs, as well as secondary bile acids (secondary BAs), which are studied in relation to the regulation of both human obesity and the intestinal microbiome [42,43].

### 3.1. Short-Chain Fatty Acids

SCFAs are organic acids with the chain length < six carbons [13]. They are predominantly produced by the anaerobic bacterial fermentation of indigestible food components, such as dietary fiber (DF) and resistant starch (RS; [20]), which are also collectively referred to as microbiota-accessible carbohydrates (MACs; [44,45]).

The most represented SCFAs in the human colon are acetate (60% of total SCFAs), propionate (20%), and butyrate (20%; [46]).

One of the SCFAs’ functions in the gut is to strengthen integrity and modulate the permeability of the gut barrier [13]. Butyrate upregulates the expression of the genes coding for claudin-1 (CLDN-1), zonula occludens-1 (ZO-1), and occludin (OCLN), proteins composing tight junctions [47]. Butyrate also increases the expression of mucin 2, strengthening the mucus layer of the gut epithelium [48].

Butyrate is also a primary energy source (70%) for colonocytes [49]. SCFAs stimulate secretion of glucagon-like peptide 1 (GLP-1) and peptide YY (PYY) and, as such, participate in the regulation of appetite [50] and reduction of body weight [51]. Due to the suppression of hepatic gluconeogenesis (by propionate; [52]) and lipogenesis (by acetate and butyrate; [53]) modulate SCFAs both glucose and lipid metabolism. SCFAs are also able to activate the G protein-coupled receptor GPR41 and stimulate production of leptin [54].

**Figure 1:**
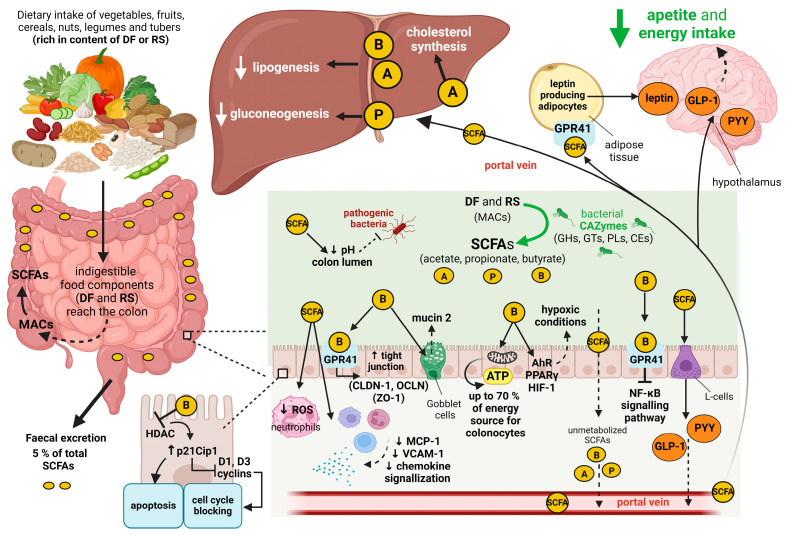
Formation of short-chain fatty acids and their effects on the human organism. DF, dietary fiber; RS, resistant starch; MACs, microbiota-accessible carbohydrates; SCFAs, short-chain fatty acids; A, acetate; P, propionate; B, butyrate; CAZymes, carbohydrate-active enzymes; GHs, glycoside hydrolases; GTs, glycosyltransferases; PLs, polysaccharide lyases; CEs, carbohydrate esterases; GPR41, G protein-coupled receptor 41; ROS, reactive oxygen species; MCP-1, monocyte chemoattractant protein 1; VCAM-1, vascular cell adhesion molecule 1; CLDN-1, claudin-1; OCLN, occludin; ZO-1, zonula occludens-1; ATP, adenosine triphosphate; AhR, aryl hydrocarbon receptor; PPARγ, peroxisome proliferator-activated receptor gamma; HIF-1, hypoxia-inducible factor 1; NF-κB, nuclear factor kappa B; GLP-1, glucagon-like peptide 1; PYY, peptide YY; HDAC, histone deacetylase; p21Cip1, cyclin-dependent kinase inhibitor 1; D1 and D3, cyclins 1 and 3. Created with Biorender.com.

The impact of SCFAs on innate and adaptive immunity is based, among other things, on decreased production of reactive oxygen species (ROS) in neutrophils [55] and on suppressed expression of monocyte chemoattractant protein 1 (MCP-1), vascular cell adhesion molecule 1 (VCAM-1), and chemokine signaling [56,57]. Moreover, butyrate is able to suppress the signaling pathway of nuclear factor kappa B (NF-κB), thereby reducing gut inflammation [58]. The formation of SCFAs and their effects on the host organism are summarized in Figure 1.

Species within Firmicutes families Lactobacillaceae, Ruminococcaceae, and Lachnospiraceae produce SCFAs by breaking down complex polysaccharides [59]. Bifidobacteria species such as *B. bifidum*, *B. infantis*, and *B. breve* dominate the infant gut microbiome and utilize human milk oligosaccharides, producing predominantly acetate [60]. Firmicutes, especially in the Clostridia class, start to increase when more diverse food is available [61], increasing the gut concentration of propionate [62].

As far as butyrate is concerned, some species belonging to the Actinobacteria (especially *Bifidobacterium* spp.), Fusobacteria, and Proteobacteria phyla are able to generate butyrate [63], with most butyrate-producing bacteria belonging to the phylum Firmicutes [13]. Species of the families Lachnospiraceae and Ruminococcaceae are important butyrate producers [64,65], but one of the most abundant butyrate-producing bacteria, representing up to 5% of the colon microbiota of healthy adults, is *Faecalibacterium prauznitzii*, a member of the Ruminococcaceae family [66,67].

For maintaining a healthy intestinal environment, butyrate-producing bacterial communities are instrumental [13]. The most frequently used strategies for promoting growth of butyrate-producing bacteria include consumption of probiotics (selected strains of *Bifidobacterium* spp., especially in dairy products) and/or prebiotics (see Section 5.1). The consumption of specific prebiotics, such as inulin-type fructans and arabinoxylan-oligosaccharides, leads to cross-feeding interactions between Bifidobacteria and other butyrate-producing bacteria (predominantly *F. prauznitzi*, *Anaerostipes*, *Eubacterium* and *Roseburia* species) with a butyrogenic effect [68].

The production of butyrate as the substrate for the generation of energy for colonocytes enables these epithelial cells to increase oxygen consumption and maintain an anaerobic environment in the gut [11,69]. As a consequence, the growth of the opportunistic aerobic pathogens such as *Salmonella* and *Escherichia coli* can be suppressed [70].

SCFAs produced by the gut microbiota are rapidly absorbed by the colonic epithelium and can be used by the epithelial cells themselves (butyrate is a primary energy source for the colonocytes) or within the host metabolism. Acetate is engaged in cholesterol synthesis and, as a precursor for the biosynthesis of long-chain fatty acids, in lipid metabolism [19]. Propionate is, among other things, a substrate for gluconeogenesis in the liver and thus contributes to glucose homeostasis. As a part of the gut–brain axis, SCFAs (especially propionate and acetate) modulate production of the host appetite-regulating hormones and, consequently, satiety and energy intake [21]. Butyrate, except for when acting as the above-mentioned energy source for gut epithelial cells, participates in the regulation of gene expression in these cells as an inhibitor of histone deacetylase (HDAC) with the possible consequence of stimulating the apoptosis and therefore decreasing risk of colorectal cancer [71,72].

As far as the relationship between SCFAs and obesity is concerned, most experiments and meta-analyses report increased fecal SCFAs in obese individuals compared to the healthy lean individuals [73,74]. The main mechanisms of increased concentration of SCFAs in stool of obese people may be: (1) increased bacterial production and shifts in cross-feeding between bacterial producers of SCFAs; (2) disorders of mucosal absorption; or (3) speed of consumed food transit [75]. Additionally, serum and plasma levels of propionate and butyrate may be reduced in individuals with a higher BMI [76,77]. However, current knowledge of changes in blood SCFA concentrations during the development and progression of obesity is incomplete because most studies deal with the measurement of fecal SCFAs concentration in the stool, where SCFAs are more detectable [74].

### 3.2. Bile Acids

Sensu stricto, only secondary bile acids (secondary BAs) are products of the gut microbiota metabolism; primary bile acids (primary BAs) are synthetized in the host liver and are mentioned in the following text only as precursors of secondary bile acids.

**Figure 2:**
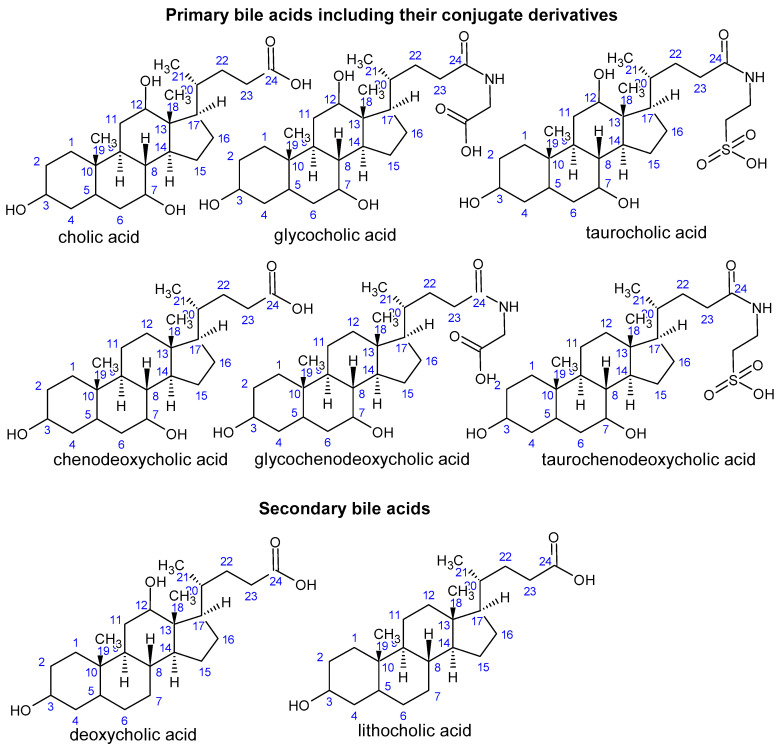
Structure of quantitatively and qualitatively important primary and secondary bile acids.

A molecule of a human bile acid (BA) includes 24 carbons forming a four-ring steroid nucleus (three six-membered rings A, B and C, and one five-membered ring D), an aliphatic side chain and two methyl groups attached at the C-18 and C-19 positions of the main carbon skeleton ([16]; Figure 2). Human primary BAs comprise cholic acid (CA; 3α,7α,12α-trihydroxy-5β-cholan-24-oic acid) and chenodeoxycholic acid (CDCA; 3α-7α-dihydroxy-5β-cholan-24-oic acid), together with their respective amino acid conjugates; the lipophilic CA and CDCA are conjugated in the liver with taurine and glycine to the more hydrophobic taurocholic acid (TCA) and taurochenodeoxycholic acid (TCDCA), and glycocholic acid (GCA) and glycochenodeoxycholic acid (GCDCA), respectively. Human secondary BAs consist of deoxycholic acid (DCA; 3α,12α-dihydroxy-5β-cholan-24-oic acid) and lithocholic acid (LCA; 3α-hydroxy-5β-cholan-24-oic acid; Figure 2). The amphipathic nature of BAs is given by the hydrophilic hydroxyl and carboxyl groups and hydrophobic methyl groups, respectively, possessing surface activity with an ability to form micelles [78].

After food intake, enteroendocrine cells of the duodenum produce hormone cholecystokinin that stimulates the secretion of conjugated primary BAs from the gallbladder into the small intestine [79] where these BAs facilitate the digestion and absorption of dietary lipids, cholesterol, lipophilic vitamins, and other hydrophobic food components due to their surface-active properties [80].

Conjugated primary BAs, which are secreted to the duodenum and proceed through the small intestine, are reabsorbed in the terminal ileum by the apical sodium-dependent BA transporter (ASBT) and subsequently transported back to the liver within the enterohepatic circulation [81]. A small amount of the primary BAs proceed to the colon, where these BAs are converted to the more hydrophobic secondary BAs (DCA and LCA). The secondary BAs are reabsorbed from the colon passively. Altogether 95% of BAs are reabsorbed from the ileum and colon through the portal vein [82].

The major regulator of the BAs’ synthesis, transport, secretion, and absorption is farnesoid X receptor [83]. After BAs ligation, FXR dimerizes in enterocytes with the retinoid X receptor (RXR), activating transcription of the gene coding for proteins performing transcellular transport of BAs [84]. The data of Friedman et al. [85] indicate that the FXR activation modulates the gut microbiota composition via the BAs-dependent mechanism, suppressing BAs synthesis in the liver and stimulating the proliferation of the Gram-positive species, such as *Streptococcus thermophilus*, *Lactobacillus casei*, *Lactobacillus paracasei*, *Bifidobacterium breve* or *Lactococcus lactis.*

Conjugated BAs are also able to bind to dietary fiber (DF). Diets rich in whole grains significantly increase concentrations of primary conjugated BAs taurocholic, glycocholic, and taurolithocholic acids with a possible resultant activation of FXR and Takeda G-protein-coupled receptor 5 (TGR5), affecting glucose homeostasis [86].

During their intestinal transit, bound primary (conjugated) BAs undergo further structural modifications and transformations to secondary BAs by the gut microbiota, after previous deconjugation and subsequent 7α-dehydroxylation [87,88].

Deconjugation reactions are catalyzed by the microbial bile salt hydrolases (BSHs) encoded by the *bsh* genes that are present in all major phyla of the gut bacteria (Firmicutes, Bacteroidetes, Actinobacteria, Proteobacteria), including gut archaea [89]. Bacterial genera capable of cleaving off the amino acid (taurine, glycine) moiety comprise Gram-positives *Bifidobacterium*, *Lactobacillus*, *Clostridium*, *Enterococcus*, and *Listeria*, but also Gram-negatives, such as *Stenotrophomonas*, *Bacteroides*, and *Brucella* [90]. Deconjugation is a necessary first step for further modifications [89].

Deconjugated primary BAs, as signaling molecules, are able to modify the total BAs pool. Because deconjugation increases amounts of antimicrobially effective BAs (CA, CDCA), the gut microbiota is supposed to evolve the deconjugation mechanism as a means of competition; deconjugation is therefore considered an essential function of the gut bacteria [90]. Due to the fact that all major phyla of the gut bacteria are endowed with enzymes able to catalyze deconjugation reactions, horizontal transfer of the genes coding for these enzymes is supposed [91].

Deconjugated primary BAs undergo 7α-dehydroxylation just by several bacterial species such as *Clostridium scindens*, *C. hylemonae*, and *C. hiranonis* (now classified as *Peptacetobacter hiranonis*; [92]). Although many bacteria are able to perform dehydroxylation reactions, only enzymes inducing 7α-dehydroxylation encoded by the genes located in the *bai* operon of the above-mentioned bacteria lead to the formation of secondary BAs [93], specifically deoxycholic acid (DCA) from CA and lithocholic acid (LCA) from CDCA, respectively, which are the most abundant secondary BAs in humans [15].

**Figure 3:**
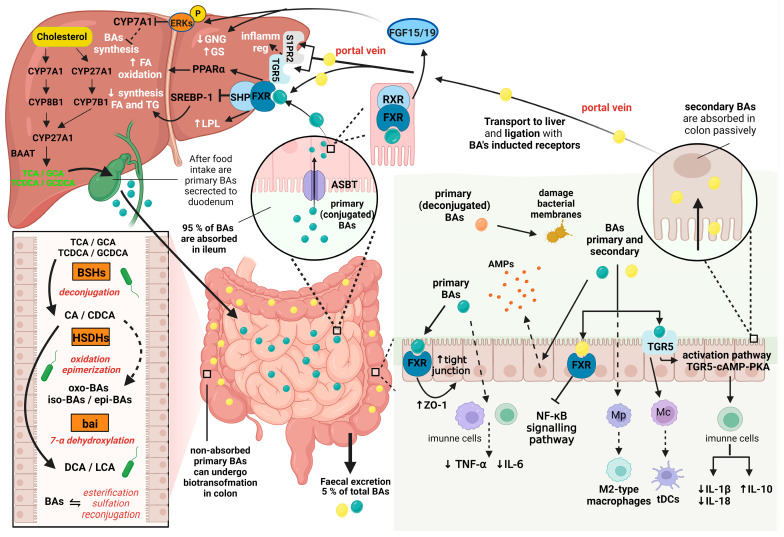
Summary of the synthesis, reabsorption, biotransformation, and physiological effects of bile acids in the human body. Abbreviations: CYP7A1, cholesterol 7α-hydroxylase; CYP8B1, sterol 12α-hydroxylase; CYP27A1, sterol 27α-hydroxylase; CYP7B1, 25-hydroxycholesterol 7α-hydroxylase; BAAT, bile acid-CoA:amino acid N-acyltransferase; TCA, taurocholic acid; GCA, glycocholic acid; TCDCA, taurochenodeoxycholic acid, GCDCA, glycochenodeoxycholic acid; CA, cholic acid; CDCA, chenodeoxycholic acid; DCA, deoxycholic acid; LCA, lithocholic acid; BAs, bile acids; BSHs, bile salt hydrolases; HSDHs, hydroxysteroid dehydrogenases; bai, BA-inducible bai operon; ASBT, apical sodium-dependent bile acid transporter; FXR, farnesoid X receptor; ZO-1, zonula occludens-1; TNF-α, tumor necrosis factor alpha; NF-κB, nuclear factor kappa B; AMPs, antimicrobial peptides; TGR5, Takeda G protein-coupled receptor 5; Mp; macrophages; Mc, monocytes; tDCs, tolerogenic dendritic cells; TGR5-cAMP-PKA, TGR5-cyclic adenosine monophosphate-protein kinase A; IL-1β, IL-6, IL-10, IL-18, interleukin 1 beta, 6, 10, 18; RXR, retinoid X receptor; FGF15/19, fibroblast growth factor 15 and 19; P, phosphorylation; ERKs, extracellular signal-regulated kinases; GNG, gluconeogenesis; GS, glycogen synthesis; SHP; small heterodimer partner; LPL, lipoprotein lipase; SREBP1, sterol regulatory element-binding protein 1; PPARα, peroxisome proliferator-activated receptor alpha; FA, fatty acids; TG, triglycerides; S1PR2, sphingosine-1-phosphate receptor 2; inflamm reg, inflammation regulation. Created with Biorender.com.

Bacterial taxa mediating bile acid biotransformation reactions are summarized in Table 1; an overview of the synthesis, reabsorption, biotransformation, and physiological effects of BAs in the human body is shown in Figure 3.

From the physicochemical viewpoint, BAs are detergents acting on the membrane of enterocytes and enabling the absorption of fatty acids in the small intestine. For the same reason, BAs are also able to disrupt bacterial membranes. However, the effect differs between conjugated and non-conjugated BAs. Primary BAs conjugated with glycine or taurine are fully ionized at physiological pH, which prevents them from significantly interacting with bacterial membranes. On the other hand, non-conjugated BAs (CA, CDCA) are able to disrupt bacterial membranes and damage the bacterial cell [94]. Sannasiddappa et al. [95] and Kurdi et al. [96] reported that non-conjugated BAs decreased the viability of the tested bacterial species (*Staphylococcus aureus*, *Lactobacillus* spp., *Bifidobacterium* spp.) more than their conjugated counterparts. From this viewpoint, deconjugation can be seen as a means of competition between gut bacteria and is it an essential function of the gut microbiota [90].

BAs composition and pool size exert antimicrobial effects both directly, by their detergent properties, and indirectly, via stimulating the production of antimicrobial peptides (AMPs) and modulating the host immunity [97,98]. BAs also modulate microbiome composition; CA supports but DCA inhibits abundances of the species of the Firmicutes phylum, DCA, on the other hand, stimulates the proportion of Bacteroidetes.

**Table 1:** Overview of bacterial taxa mediating bile acid biotransformation reactions.

Biotransformation Reactions	Phylum	Genera	Reference
deconjugation	Bacteroidetes	Bacteroides Parabacteroides Barnesiella Alistipes	[39,99]
	Firmicutes	Clostridium Eubacterium Ruminococcus Lachnospira Roseburia Lactobacillus Enterococcus	[14,15,99,100]
	Actinobacteria	Bifidobacterium Eggerthella	[15,99]
7-α dehydroxylation	Firmicutes	*Clostridium* spp. (*C. scindens*) (*C. hylemonae*) Peptacetobacter hiranonis	[14,92,101]
oxidation/epimerization	Firmicutes	Clostridium Eubacterium Collinsella	[14]
	Bacteroidetes	Bacteroides	
sulfation	Firmicutes	Clostridium Fusobacterium Peptococcus	[15]
	Proteobacteria	Pseudomonas	
esterification	Firmicutes	Eubacterium Lactobacillus	[15,102]
	Bacteroidetes	Bacteroides	

BAs concurrently modulate intestinal barrier function and immune homeostasis [15]. Primary BAs improve intestinal barrier function by inducing the expression of zonula occludens 1 (ZO-1) and concurrently decreasing the expression of tumor necrosis factor alpha (TNF-α) and interleukin 6 (IL-6; [103]). As FXR agonists, primary BAs stimulate the expression of the tight junction proteins [104], contrary to secondary DCA that impairs BAs deconjugation, thus inhibiting FXR activation with a resulting impairment of the mucosal barrier function [105]. On the other hand, DCA also stimulates expression and secretion of human β-defensins 1 and 2, improving maintenance of intestinal homeostasis [106]. Moreover, BAs promote epithelial regeneration via TGR5 signaling [107].

As far as BAs’ effects on intestinal innate and adaptive immunity are concerned, the BAs’ stimulation of TGR5 promotes differentiation of monocytes into tolerogenic dendritic cells [108] and activation of FXR inhibits signaling pathway of NF-κB [109]. Moreover, BAs promote polarization of macrophages toward the anti-inflammatory phenotype M2 [110] and activate the TGR5-cAMP (cyclic adenosine monophosphate)-PKA (protein kinase A) signaling pathway, thus limiting IL-1β and IL-18 and stimulating IL-10 production [111].

Methods of BAs analysis were recently summarized by Singh et al. [16]. Currently, the most widely used methods of BAs analysis include GC/MS and LC/MS, especially HPLC/UHPLC (high-performance liquid chromatography/ultra-high-performance liquid chromatography) with a tandem mass spectrometry (MS/MS; [112]).

## 4. Relationships Between Diet and Obesity

Before dealing with the relationship between the host diet and the gut microbiota (Section 5), it could be useful to briefly mention relationships between diet and obesity in general.

Diet composition and an increased energy intake with low physical activity (reduced energy expenditure) are the key factors leading to an increased prevalence of obesity [113]. Origin and progression of obesity can be explained using the energy balance model (EBM) and/or the carbohydrate-insulin model (CIM) [114].

According to the EBM model, the brain (hypothalamus) regulates body weight via controlling food intake through complex internal endocrine, metabolic, and nervous system signals from peripheral organs alongside external signals from the food environment [115]. The increased availability of ultra-processed foods (UPF) with high energy density, high fat and sugar content, but low protein, fiber [116], vitamin and mineral content [117] can lead to a positive energy balance and accumulation of body fat (adiposity), regardless of the diet macronutrient composition [118]. Primary examples of UPF are refined cereals, sweet/savory snacks, margarine, reconstituted and ready-to-eat frozen food or carbonated and alcoholic beverages [117]).

The CIM model puts emphasis not on the quantity, but rather on the quality (chemical composition) of the diet [119]. UPF and other refined foods contain excessive sugars, which increase glycemic index and glycemic load [118]. Increased glycemia stimulates an overproduction of insulin, which leads to an increased adiposity with concurrent suppression of the energy released from the adipose tissue. The postprandial lack of energy substrates in blood is sensed by the hypothalamus, resulting in appetite stimulation and reduced energy expenditure, which can lead to a positive energy balance due to the hyperphagia [120].

Excessive consumption of UPF can also influence gut microbiome [121] and is likely associated with an increased prevalence of obesity and a higher risk of developing metabolic syndrome, hypercholesterolemia, and hypertension [122].

Moreover, the EBM and CIM models are currently being combined with the reduction–oxidation model (REDOX) and the obesogens model (OBS). The REDOX model is based on modulation of the production of reactive oxygen species (ROS); OBS tries to explain the current prevalence of human obesity by the obesogens (such as saturated/trans fatty acids, fructose, food additives, drugs, or insecticides) present in the diet or entering the organism from the environment [119].

Based on the data of several meta-analyses, low-carbohydrate and low-fat diets have significant potential for weight reduction [123,124]. High-protein diets, applied usually with dietary carbohydrate or fat restriction, can also be effective for the weight loss via stimulation of satiety and induced thermogenesis (energy expenditure). Regular protein intake is also necessary to preserve the muscle mass [125,126].

Apart from essential macronutrients, the intake of DF is also important for the prevention of obesity and regulation of excessive weight. DF, aside from having very low energy density, stimulates satiety and reduces macronutrients absorption and thus energy intake. People on the typical obesogenic Western-type diet consume on average 15 g of DF per day, which is only half of the recommended daily intake [127].

Still, the most effective mechanism of body weight loss is a caloric deficit, regardless of the macronutrient’s composition [125,128]. Caloric restriction can also be employed in treatment of obese individuals by the dietary systems based on intermittent fasting [129]. However, it is important to mention in this context that an irregular timing of the food intake (meal frequency) can disrupt circadian rhythms, resulting in an increased body weight [125].

## 5. Host Diet and Gut Microbiota

Diet is an important factor influencing intestinal microbiota, which can have a potential therapeutic effect for the adjusting of composition, diversity, and stability of the intestinal microbiome [130]. Short-term diets and specific dietary interventions have been shown to rapidly alter gut diversity. However, these changes are often transient and do not persist after the diet and the intervention have ended [131]. Long-term eating habits and a typical diet across populations from different geographical areas correspond with microbial composition and are thus associated with the stability of a given enterotype of an individual or group of individuals [132,133]. The so-called Western-type diets tend to be lower in fiber and higher in fat and refined carbohydrates in comparison with diets used in rural areas and Eastern countries [134]. As far as geographical location is concerned, other factors, such as food availability and nutritional status, amount of physical activity, level of hygiene, or the prenatal/postnatal care and infant feeding, may influence the differences in the composition of the intestinal microbiome [135].

Diets rich in protein and fat and diets rich in carbohydrates are often associated with Bacteroides-abundant (enterotype I) and Prevotella-driven (enterotype II) microbiota profiles, respectively [10]. This follows the data of Wu et al. [132] where Bacteroidetes and Actinobacteria positively correlated with dietary fat but negatively with dietary fiber (DF); the opposite behavior was reported for Firmicutes and Proteobacteria [132]. In contrast, Brinkworth et al. [136] found that high-fat/low-fiber diets reduced the abundance of Bifidobacteria compared to low-fat/high-fiber diets. Reduced carbohydrate intake (along with reduced DF content) led to the reduction of bacteria such as *E. rectale*, *Roseburia* spp., and *Bifidobacterium* spp. in obese adults [137]. DF increased gut abundance of *Prevotella*, while bile resistant genera like *Bilophila* and *Bacteroides* correlated with a high-fat animal-based diet [131].

The abundances of dominant species of colonic microbiota can be modified rapidly and reversibly by dietary intervention, which was demonstrated by Walker et al. [138] in a cohort of overweight men on a diet high in resistant starch (RS). Despite predominant inter-individual differences, the RS-diet rapidly changed abundances of the specific bacterial groups, with the most conspicuous increase in *Ruminococcus bromii* and *Eubacterium rectale*; however, the changes were rapidly reversed by the subsequent diet. De Filippo et al. [139], when comparing the diets of children from rural Africa and Europe, respectively, found that fiber-rich diets stimulate genera within the Bacteroidetes phyla (specifically *Prevotella* and *Xylanibacter*), but animal-based and sugar-rich diets increase amounts of Firmicutes and Enterobacteriaceae.

Vegetarians generally tend to have higher bacterial diversity and higher ratios of *Prevotella* to *Bacteroides* and reduced numbers of Enterobacteriaceae, including *Escherichia coli*, compared to omnivores [140]. Compared to omnivores, vegetarians and vegans also have a higher abundance of Lachnospiraceae (genera *Roseburia*, *Anaerostipes*, *Blautia*) and Ruminococcaceae (genera *Ruminococcus* and *Faecalibacterium prauznitzii*), and a lower abundance of *Bacteroides*, *Parabacteroides*, and *Alistipes* [141].

The intake of vegetable proteins based on whey or peas can increase the abundance of the genera *Bifidobacterium* and *Lactobacillus* [142,143]. On the other hand, consumption of animal proteins leads to increased abundances of *Bacteroides*, *Alistipes*, and *Bilophila*, and also some species of the genera *Clostridium* [142]. Shoonakker et al. [144] reported a decrease in the family Lactobacillaceae and Enterobacteriaceae after a dietary protein restriction. Generally speaking, bacteria with proteolytic and amino acid degrading activity comprise genera *Clostridium*, *Fusobacterium*, *Peptostreptococcus*, *Veillonella*, and *Bacteroides* [145]. However, it should be mentioned that controlled human studies exclusively examining the effect of different dietary protein sources are limited and most of the evidence comes from model animal studies [146].

As far as dietary lipids are concerned, the results of intervention studies show reduced bacterial diversity owing to the high-fat diet (HFT) and reduced total numbers of resident bacteria, including Firmicutes [143]. On the other hand, HFT typically increases genera such as *Alistipes* and *Bacteroides*, while dietary fat restriction leads to increased numbers of beneficial *Faecalibacterium* [144,147].

The Western-type diet, characterized by high protein and fat (especially saturated fat) intake are associated with higher incidence of metabolic disorders (T2D), cardiovascular diseases and obesity [148,149], increases abundances of *Bacteroides*, *Alistipes*, and *Bilophila*, and decreases genera *Lactobacillus*, *Roseburia*, *Eubacterium* and *Enterococcus* [150,151]. In contrast, the Mediterranean diet, based on dietary fiber (cereals, legumes, vegetables, fruits, and nuts), unsaturated fatty acids (fish, vegetable oils) and antioxidants (flavonoids, polyphenols; [152,153,154]), increases overall microbial diversity, including families of Clostridiaceae and Lactobacillaceae and specifically genera such as *Bacteroides*, *Bifidobacterium*, *Prevotella*, *Roseburia*, *Clostridium*, *Lactobacillus*, and *Faecalibacterium*, and decreases abundances of Proteobacteria [153,155,156,157].

Boosting fiber consumption to 40 g/day in healthy adults increased fecal abundance of a group of Lachnospiraceae (ND3007), a SCFA-producing family of bacteria with a concomitant tendency to increase the butyrate-producing capability [158].

### 5.1. Dietary Fiber

Dietary fiber (DF) is possible to subdivide into insoluble dietary fiber, including prebiotics, and soluble dietary fiber [17]. Whole wheat flour, brown rice, nuts, beans, and vegetables [159] belong to the main sources of insoluble dietary fiber (cellulose, hemicelluloses and fructans; [160]). Prebiotics, a special class of insoluble fiber, are defined as “non-digestible compounds that, when consumed, induce changes in composition and/or activity of the gastrointestinal bacteria, thus causing benefit(s) upon host health” [161].

Prebiotics are classified based on their ability to withstand passage through the gastrointestinal tract (resistance to gastric acidity and to the host hydrolytic enzymes, gastrointestinal absorption), on their suitability for fermentation by intestinal microbiota, and on their ability to stimulate the growth and activity of intestinal bacteria with positive effects on the host health [162]. Selective fermentation, modulation of intestinal pH, bulk of stool, growth inhibition of pathogenic bacteria, and host protection against toxic metabolites of the putrefactive bacteria are usually listed as the principal attributes of prebiotics. Fructooligosaccharides, galactooligosaccharides, xylooligosaccharides, arabinooligosaccharides, oligofructose, inulin, β-glucan, guar gum, resistant starch, and maltodextrin are considered most important types of prebiotics [17].

Soluble fiber, contrary to insoluble fiber, are viscous gel-like substances able to slow absorption of nutrients in the intestine [163]. Pectin, guar gum, and some types of inulin, present in whole grains, legumes, seeds and nuts, and some fruits and vegetables are the most widely used representatives of soluble fiber [164].

The positive effects of DF on the gut microbiome and on the gut epithelium include production of SCFAs [159], such as SCFAs-mediated modulation of the epithelial barrier functions [165] and the host innate immune defenses [166]. Therefore, one of the possible dietary interventions for obesity is to support the gut with carbohydrate-associated enterotype [10] by securing the sufficient intake of DF, including also resistant starch [6].

From this viewpoint it is necessary to view the gut microbiota as a hugely diverse ecosystem [18]. As far as diversity is concerned, alpha diversity (within-community diversity) and beta diversity (measure of similarity between communities) is usually used when diversity indicators are calculated [167]. Richness (number of taxonomic groups), evenness (taxa abundances distribution), and phylogenetic diversity (phylogenetic distance regarding the branch lengths in the phylogenetic tree) are indispensable tools as far as alpha diversity metrics are concerned [168].

The intake of DF is able to rapidly change the composition of the human gut microbiome [169]. However, dietary interventions using fermentable DF aiming to promote gut alpha diversity are often ineffective and their results are contradictory, as shown in a meta-analysis of pertinent clinical trials [170]. Moreover, Zhang et al. [171] demonstrated alleviation of children obesity with concomitantly reduced gut alpha diversity.

As far as the above-mentioned richness is concerned, it can be increased by a long-term intake of DF [172]. Wang et al. [158] even reported higher fecal alpha diversity in a short-term (2 weeks) two-phase randomized cross-over trial swapping white bread for high-fiber bread (fiber intake 40 g/day). However, DF intake is mostly associated with a reduction in richness of the gut microbiota [173,174]. The likely reason is related to the substrate-specific abilities of the gut microbiota to utilize the available substrates [175]. Therefore, limited overall substrate availability or changing the type of substrate can cause loss of gut bacteria and reduce richness [176].

Changes in the parameter of evenness (equitability of the proportions of the bacteria composing a given community) are dependent on the abundances of bacteria sharing the fiber substrate and on the initial composition of the gut microbiome [177]. Therefore, decreased evenness within the highly competitive gut environment after supplementation of different types of DF [176] can be explained by a greater chance of the initially highly abundant bacteria (provided they are the substrate utilizers), as compared to the low abundant bacteria to utilize the supplemented substrate [177]. Consequently, the last-mentioned authors Cantu-Jungles and Hamaker [177] conclude that despite still achieving health-related improvement after the fiber supplementation, reduction in alpha diversity is possible, even expected.

In order for DF to provide health-beneficial metabolites in the gut, microbial communities must be present that are able to express specific genes coding for enzymes that degrade and metabolize complex carbohydrates [178]. These so-called carbohydrate-active enzymes (CAZymes; [179]) can be divided into several classes, such as glycoside hydrolases (GHs), glycosyltransferases (GTs), polysaccharide lyases (PLs), carbohydrate esterases (CEs), and carbohydrate-binding modules [180]. As an example of a relatively versatile fiber-utilizing genera, *Bifidobacterium* has at its disposal CAZymes for metabolizing arabinoxylan-derived oligosaccharides and inulin-type fructans [181,182]. On the other hand, *Monoglobus pectinilyticus* is a highly specialized pectin degrader whose genes code for special CEs and PLs [183]. *Lacticaseibacillus paracasei* was able to express an extracellular exo-inulinase in the presence of a β-(2,1)-fructan, which increased availability of fructooligosaccharides for other bacteria [184].

Supplementation by dietary fiber rich in arabinans and galacturonans increased the pool of genes encoding for arabinofuranosidases, β-glucanases, β-xylanases, and polysaccharide lyases [185]. Rivière et al. [68] demonstrated the importance of the cross-feeding reactions between *Bifidobacterium* and the butyrate producing genera *Roseburia* and *Anaerostipes* for the production of the host-beneficial metabolites.

Kok et al. [18] conclude that fiber utilization in the human gut can be successfully realized either as a particular bacteria-substrate interaction or via cross-feeding interactions with participation of several taxa. However, for a successful fiber utilization, not only are the above-mentioned CAZymes necessary, but also regulatory elements, transporters, and binding proteins comprising carbohydrate gene clusters [179]. Following the discovery of the starch utilization system in *Bacteroides thetaiotaomicron*, the glycan utilization system called polysaccharide utilization loci (PULs) was characterized in genera *Bacteroides* in general [186]. On the other hand, species within Firmicutes and Actinobacteria degrade glycans using various transport systems, and the presence of PULs encoding for polysaccharide-degrading enzymes, oligosaccharide transporters, and transcriptional regulators were reported in strains of the Firmicutes phylum [187].

According to Kok et al. [18], a response of the gut microbiome to DF supplementation is highly personalized. Rodriguez et al. [188] distinguished, after 3 months of inulin supplementation in a cohort of obese patients, a subgroup of responders (BMI decrease), showing higher abundances of *Akkermansia* and *Butyricicoccus* and a lower abundance of *Anaerostipes*. Therefore, though Kok et al. [18] admit a possibility for a modulation of the gut microbiome by dietary interventions, the authors concurrently recommend employing microbiome-derived biomarkers for the subjects’ classification [18]. Chen et al. [189] proposed an employment of enterotypes, namely Prevotella-, Bacteroides- and Ruminococcus-dominant enterotypes, in this context. Treated with a high-fiber diet, individuals with Prevotella enterotype reduced weight more efficiently than individuals with Bacteroides enterotype [190]. Similarly, better fiber-utilizing ability, including a higher SCFAs concentration, was reported with Prevotella enterotypes as compared to Bacteroides enterotypes [189].

One of the suitable biomarkers regarding fiber metabolism is *Bifidobacterium* spp., an example of a single taxonomic enrichment after supplementation of the prebiotic fibers [191]. Kok et al. [18] summarized results of the studies differentiating responders and non-responders by observing enrichment of *Bifidobacterium* spp. after providing different types of DF, including galactooligosaccharides or agave inulin. Responders, as compared to non-responders, were often individuals with less stable gut microbiome [18,192]. Higher abundances of Bacteroidetes and Firmicutes at baseline were reported in responders and non-respondents, respectively [18]. Magne et al. [193] suggested a ratio of Firmicutes/Bacteroidetes as a suitable marker for evaluating gut dysbiosis in obese patients.

However, effects of the fiber-rich dietary interventions based exclusively on the enterotype-dominance should be considered with caution in the general population due to confounding factors such as a baseline diet, exercise, or use of antibiotics [194]. Probands classified as high-dietary fiber consumers at a baseline, in comparison with low-dietary fiber consumers, showed increased abundances of *Bifidobacterium* and *Faecalibacterium* and decreased amounts of *Coprococcus*, *Dorea*, and *Ruminococcus* after dietary inulin intervention, demonstrating a broader inulin-conditioned taxonomic response [195].

### 5.2. Resistant Starch

Starches in general are macromolecules based on glucose, stored in many plants as a primary source of energy [196]. However, from the viewpoint of providing energy to the human organism, a substantial difference exists between various types of starches. Most types of starch are easily hydrolyzed in the small intestine by human saccharolytic enzymes (α-amylase, maltase and isomaltase; [197]). On the other hand, resistant starch (RS) escapes hydrolysis in the small intestine and proceeds undigested into the large intestine, where they are broken down by enzymes produced by the gut microbiota [198].

The glucose units in starches are arranged in linear chains of amylose (α-1,4-glycosidic linkage) and a branched-chained amylopectin (α-1,6-glycosidic bonds; [199]). The digestibility/resistance of the starches is primarily determined by the amylose/amylopectin ratio and their organization within starch granules. The more densely and more tightly packed these components are in the starch granules, the less accessible they are to the human digestive enzymes [200].

Grains (whole grains of barley, oats, and wheats), legumes (lentils, chickpeas, beans), tubers (potatoes and yams; their cooking and subsequent cooling induces starch retrogradation), and some kinds of the processed foods (whole grain breads, pasta underwent to the extrusion cooking) belong to the most important food sources of RS.

According to their origin and main properties, RS is usually divided into four types, RS type 1 to RS type 4 [19]. RS type 1 (RS1) is physically inaccessible to the digestive enzymes due to a specific polysaccharide-protein structure forming the protective barrier. Whole grains and seeds are the common sources [201]. The native granular form of certain raw foods, such as raw potatoes or green bananas, is typical for RS type 2 (RS2). The tightly packed granular structure of amylose, present in high concentration in RS2, limits access of digestive enzymes [202]. The above-mentioned cooking and subsequent cooling of certain food matrices (tubers) elicits realignment and recrystallization of the starch molecules that became resistant to saccharolytic enzymes. Cooked and cooled potatoes, pasta and rice thus contain considerable amounts of this RS type 3 (RS3; [198]). Chemically modified starches not naturally occurring in foods are categorized as RS type 4 (RS4). Their resistance to digestion is mainly based on the cross-linking of the starch macromolecules introduced by various industrial processes [203].

Particular microbial taxa or the species within the taxa might prefer a certain type of RS consumed by the host. A typical example is a predilection of *Ruminococcus bromii* for RS2 from high-amylose maize or of some *Bacteroides* species for RS3 from retrograded starch [19]. Accessibility and fermentability of RS can be influenced not only by the presence of other types of dietary fibers in the host gut, but also by intake of basic nutrients (proteins, lipids) and micronutrients [204].

As an example, diets rich in soluble fibers can increase the abundance of *Lactobacillus* spp., supporting the positive prebiotic effect of RS. On the other hand, a protein-rich diet might steer colonic bacteria to protein fermentation with a negative outcome of ammonia production. Another important factor affecting gut microbiota composition is the duration of the RS intake. One-time consumption of RS can shift the colonic microbiota abundances relatively rapidly. However, an establishment in the gut of the microbial community more diversified and thus more resilient and more resistant to dysbiosis can be achieved by a long-time RS intake [19].

Similarly to dietary fiber (see Section 5.1), RS is hydrolyzed by saccharolytic bacteria metabolizing complex carbohydrates and subsequently producing SCFAs, mainly acetate, propionate, and butyrate [205]. Though both Bacteroidetes and Firmicutes are able to produce these volatile fatty acids, representatives of the Bacteroidetes phylum produce predominantly propionate, a species within the Firmicutes phylum that are more specialized to butyrate production, with *Faecalibacterium prauznitzii* and *Eubacterium rectale* being the principal producers [206].

As far as mechanisms of RS utilization in the colon are concerned, most important RS fermenting bacteria belong to Bacteroidetes and Firmicutes phyla [138]. *Bacteroides* spp. within the Bacteroidetes phylum or *Ruminococcus* spp. within the phylum of Firmicutes are especially proficient in breaking down RS (among other complex carbohydrates) to produce SCFAs. *Ruminococcus bromii*, in particular, is instrumental in the first stages of RS degradation enabling other microbiota constituents to access the degradation products for further fermentation [19].

The actinobacteria phylum also contributes to RS fermentation in the colon, namely the genera of *Bifidobacterium* efficiently degrades RS to produce SCFAs, resulting in the lowering of the colon pH, which renders this environment unfavorable for pathogenic bacteria [207].

Bacteria is not the only domain engaged in the RS fermentation in the colon. *Methanobrevibacter smithii*, a methanogenic member of the domain Archaea, is able to incorporate hydrogen produced by other microorganisms during RS fermentation into methane, preventing hydrogen accumulation and suppression of the fermentation processes in the gut.

The most frequently mentioned positive effects of RS on gut relate to gut barrier function, modulation of inflammation, and function of the gut immune system [19]. RS supports colonocyte turnover, thereby enhancing the epithelial barrier. RS fermentation products, SCFAs, contribute to upregulation of the expression of the tight junction proteins that are essential for maintaining the gut barrier integrity [208]. As far as modulation of inflammation is concerned, butyrate inhibits production of TNF-α and IL-6, pro-inflammatory cytokines contributing significantly to propagation of the inflammatory processes [209]. Moreover, butyrate positively affects immune cell differentiation, especially differentiation of naïve T-cells into regulatory T-cells (Tregs). Tregs are instrumental in maintaining gut immune homeostasis [210].

The three main types of current approaches for RS determination comprise enzymatic, chromatographic, and spectroscopic methods [19]. Megazyme resistant starch assays, an example of the advanced enzymatic techniques, simulates processes within the human digestive tract by an application of a series of relevant enzymes that remove accompanying components in the food sample matrix; the remaining RS is quantified spectrophotometrically. The method provides data useful in nutritional labelling [211].

Chromatographic (high-performance liquid chromatography) separation and determination of the sample components after preceding enzymatic hydrolysis is able to measure molecular size and structure and thus distinguish RS from other DF [212]. As far as spectroscopic methods are concerned, nuclear magnetic resonance spectroscopy (NMR) provides data regarding detailed RS structures on the molecular level and is therefore able to differentiate between particular types of RS [213].

## 6. Human Gut Microbiome and Obesity

Gut microbiome of the obese population is often associated with intestinal dysbiosis characterized by a loss of microbial gene richness and thus a change in composition and metabolic activities of the intestinal microbiota [214,215,216]. Dysbiosis is related to the loss of commensal bacteria, excessive growth of pathogenic/conditionally pathogenic bacteria, and loss of total microbial diversity [217,218]. For example, authors Liu et al. [219], Ciobârcă et al. [220], and Heiss and Olofsson [221] reported reduced gut microbiome diversity in obese individuals.

Obesity is often associated with an increased Firmicutes/Bacteroidetes ratio, including a low abundance of Bacteroidetes [6,222]. This finding is confirmed by Kasai et al. [223] and Koliada et al. [224] in Japanese or Ukrainian obese adult populations, respectively. Increased levels of Firmicutes and decreased abundances of Bacteroidetes in obese individuals were reported also by Ley et al. [225]. Stojanov et al. [226] reported a substantially higher likelihood of overweightness with a Firmicutes/Bacteroidetes ratio of ≥ 1 in comparison with Firmicutes/Bacteroidetes ratio of <1.

However, Breton et al. [7] point out that operating only on the level of phyla alone is imprecise. This statement is based, among other things, on findings of Cani et al. [227] which state, despite the fact that some Firmicutes genera including *Clostridium*, *Lactobacillus*, and *Ruminococcus* are increased in obesity, one of the most abundant Firmicutes species, *Faecalibacterium prauznitzii*, is decreased in this state. Similarly, an increased abundance of Bacilli and decreased abundance of Clostridia, but not an association with the Firmicutes/Bacteroidetes ratio, was found in American adults [228]. Similarly, Hu et al. [229] reported a significant association of *Bacteroides* and *Prevotella*, but not with the Firmicutes/Bacteroidetes ratio, in obese Korean adolescents. Duncan et al. [230] also reported no relationships between BMI, body weight loss, and Bacteroidetes abundance in obese and non-obese groups. Moreover, childhood obesity was associated with an increased abundance of Proteobacteria, including a positive correlation between Proteobacteria and fat intake in the study of Mendez-Salazar et al. [231].

Breton et al. [7] mention discrepancies even within particular bacterial genera. Decreased levels of *Lactobacillus paracasei* but increased abundances of *Lactobacillus reuteri* and *Lactobacillus gasseri* were found in the stool of obese subjects in comparison with lean ones [232]. So, Breton et al. [7] conclude that as far as obesity is concerned, no specific bacterial signature has been identified. Nevertheless, key obesity markers, such as increased adiposity, dyslipidemia, and insulin resistance are associated with a relatively poor gut microbiota, meaning generally low bacterial gene pool [233].

The obese enterotypes, including abundances of the gut microbiome phyla/genera/species also depend on the stage of life: Mariat et al. [234] reported Firmicutes/Bacteroidetes ratios 0.4, 10.9, and 0.6 in infants, adults, and elderly subjects. Abundances of the gut microbiota can also be rapidly changed as a consequence of dietary modifications [131].

The following factors relating gut microbiome composition to obesity can be suggested: gut microbiota-derived metabolites (SCFAs; secondary BAs), an ability of the gut microbiota to mediate low-grade inflammation [6], and effects of the gut microbiota on the intestinal immunity in obesogenic diets [9].

SCFAs produced during the fermentation of indigestible dietary substances (DF, RS) inhibit fat accumulation in adipose tissue, increase energy expenditure, and stimulate production of the satiety-inducing hormones [235], such as GLP-1 and PYY, with a consequence of decreasing weight gain [236].

One of the hallmarks of obesity is a state of chronic low-grade inflammation [237]. Bacteroidetes, as the Gram-negative bacteria, contain endotoxin lipopolysaccharide (LPS) in their cell walls that stimulates adipose tissue deposition, insulin resistance, and increased inflammation grade [238]. Trøseid et al. [239] reported elevated plasma LPS levels in obese individuals compared to healthy controls, including a significant correlation between the intra-abdominal fat volume and plasma LPS level. On the other hand, higher abundances of Gram-positive bacteria *Lactobacillus* spp. and *Bifidobacterium* spp. within the gut microbiome are able to reduce intestinal permeability and improve systemic inflammation [240,241].

Cell-mediated and humoral innate intestinal immunity consists of Goblet cells producing mucus, Paneth cells able to secrete antimicrobial peptides (AMPs), intestinal epithelial cells, innate lymphoid cells, and myeloid cells [9,242]. A direct involvement of intestinal microbiota in defensive mechanisms was demonstrated in the case of *Akkermansia muciniphila* that restored the obesogenic diet-induced downregulation of AMPs [243]. Toll-like receptors (TLRs) and NOD-like receptors (NLRs), as principal pattern recognition receptors, sense microorganisms-associated patterns (MAMPs: LPS, flagellin, peptidoglycan) and so initiate intestinal immune activation at the cellular level [9].

As far as adaptive immunity is concerned, intestinal microbiota positively modifies intestinal T-helper 17 (Th 17) cells. Th 17 cells secrete interleukin 17 (IL-17) and IL-22 that induce production of AMPs and tight junction proteins and so protect gut barrier integrity [244]. Failure of the host defense as a consequence of reduced IgA (immunoglobulin A)+ cells and decreased IgA levels in a diet-induced obesity was demonstrated by Luck et al. [245]. Obesity was related to impairment of IgA production, which was also in the study of Petersen et al. [246], where increased amounts of *Desulfovibrio* spp. and decreased abundance in *Clostridium* spp. was reported.

Gut microbiota can also affect the host immune system in obesity via structural and metabolic mediators [9]. Cani et al. [247] demonstrated the involvement of LPS from Gram-negative bacteria in obesity-associated inflammation. On the other hand, flagellin is able to ameliorate diet-induced obesity [248] and muramyl dipeptide (derived from the bacterial peptidoglycan) reduces inflammation and insulin resistance via the NOD2 receptor signaling in the study of Cavallari et al. [249]. Mazmanian et al. [250] reported induction of IL-10 in intestinal T-cells by the polysaccharide A (PSA) from the capsule of *Bacteroides fragilis*, which prevented intestinal inflammation; the immune reaction was suppressed by PSA from *Bacteroides fragilis* via activation of TLR2 on the CD4+ T-cells. TLR2 was also involved in a signaling pathway of a membrane protein from *Akkermansia muciniphila* exerting anti-obesity effects [251].

Apart from SCFAs and secondary BAs (see Section 3.1 and Section 3.2 of this study), at least two more microbiota-derived metabolites affect the host health status in obesity, including insulin resistance [9]. Imidazole propionate, a histidine metabolite, belongs to the risk factors for insulin resistance and, consequently for T2D, expansion of the imidazole propionate-producing bacteria can be elicited by dietary changes resulting to the shift in the gut environment [252]. On the other hand, immune homeostasis can be boosted by microbial metabolites produced from tryptophan [253].

The key transcription factor in this context, widely expressed in immune cells, is aryl hydrocarbon receptor (AhR). Indoles, catabolites of tryptophan, are important AhR ligands, and the gut microbiota in subjects with metabolic syndrome were reported to show a reduced ability to metabolize tryptophan into effective AhR ligands [254]. Reduced synthesis of the AhR agonists can contribute to obesity pathogenesis via decreasing production of IL-22. The gut microbiome of obese individuals shows an increased expression of indoleamine-2,3-dioxigenase, stimulating the kynurenine pathway. This leads to the degradation, instead of production, of indole derivatives and decreased concentration of IL-22, resulting in chronic inflammation and decreased insulin sensitivity [255].

## 7. Future Directions Regarding Diet, Gut Microbiome, and Obesity

The direction of further research concerning relations between diet, gut microbiome, and obesity proceed from the current knowledge of the common roots of obesity, including gut dysbiosis, due to excessive consumption and cumulative effects of high-energy food and obesogenic diets, with simultaneously reduced consumption of dietary fiber. On the contrary, lower energy intake, higher consumption of dietary fiber, and increased physical activity are likely key factors for modulation in both obesity and the gut microbiome. Moreover, the restricted intake of ultra-processed foods seems to be a suitable strategy for regulation of the gut microbiome [256]. A very important issue that is being currently addressed is the enormous intrinsic variability of the composition of the human gut microbiome. Therefore, it is necessary to modify, or even abandon, the standard dietary approaches, in a sense to move towards personalized nutrition adjusted to the physiological status and specific needs of a consumer [257]. Dietary interventions using probiotics, prebiotics, or synbiotics can be effective in regulation of the body weight. However, it should be emphasized in this context that it is necessary to perform further clinical studies for a better understanding of the selection of specific probiotics strains, frequency, optimal dose, and duration of an intervention [258]. Fecal microbiota transplantation is another potential therapeutic instrument for possible body-weight modulation. However, the reliable experimental data regarding validity of this method are still limited so further research is needed in this field [259]. Moreover, as far as the gut microbiome analysis is concerned, the traditional 16S rRNA gene sequencing is supposed to be more often superseded by the more complex shotgun metagenomics that enables profiling of entire bacterial genome, including its functional properties [142]. Creation of the more robust and comprehensive reference databases regarding composition of the gut microbiota, including their metabolites, has the potential to explain in a more detail diverse interactions within the microbiome-host system in health and disease, including obesity [260].

## 8. Conclusions

From the recent data concerning associations between gut microbiome, host diet, and obesity, it is shown that these relationships are extremely complicated, and, despite the often-quoted microbiota–gut–brain axis as a potential target for interventions, a deeper characterization of these associations is still missing. The relationships between gut microbiota and obesity are bidirectional and evidence to identify their temporal relations is still insufficient. No specific gut bacterial signature has been identified up until now as far as obesity is concerned. Moreover, gut microbiota may favour obesity via modulation of energy homeostasis, lipopolysaccharide-stimulated inflammation, and fat deposition, though it is not clear which specific bacterial communities contribute to the development of obesity. Findings regarding relationship between secondary bile acids (as products of the gut microbiota metabolism) and obesity are also still mostly inconclusive. It therefore can be concluded that further experimental effort is necessary to fulfil the potential of the gut microbiome, including products of its metabolism, SCFAs and BAs, in combination with a diet based on a sufficient amount of dietary fiber/resistant starch, to modulate obesity meaningfully.
